# Inhibition of DNA Repair Protein Ku70 in High-Glucose Environment Aggravates the Neurotoxicity Induced by Bupivacaine in SH-SY5Y Cells

**DOI:** 10.1155/2020/1283214

**Published:** 2020-01-31

**Authors:** Zhong-Hua Ji, Yu Zhou, Hui Wang, Shuang Li, Gen-qiang Liang, Jun Mao

**Affiliations:** ^1^Department of Anesthesiology, Zhuhai Hospital Affiliated with Jinan University, Zhuhai 519000, Guangdong Province, China; ^2^Department of Radiology, Zhuhai Hospital Affiliated with Jinan University, Zhuhai 519000, Guangdong Province, China

## Abstract

Bupivacaine, a common local anesthetic, causes serious nerve injury, especially in diabetic patients, as high glucose has been reported to enhance bupivacaine-induced neurotoxicity. However, the key regulator for synergism remains unknown. To our surprise, the expression of repair protein Ku70 is suppressed, while the high-glucose environment induces DNA oxidative damage in neurons. Here, we aim to investigate whether the inhibition of Ku70 by high-glucose conditions aggrandized bupivacaine-induced DNA damage. Consistent with previous results, bupivacaine induced reactive oxygen species production and upregulated Ku70 and cleaved caspase-3 expressions at both transcript and protein levels and ultimately caused nucleic acid damage and apoptosis in human neuroblastoma (SH-SY5Y) cells. High-glucose treatment inhibited the expression of Ku70 and enhanced bupivacaine-induced neurotoxicity. In contrast, the overexpression of Ku70 mitigated DNA damage and apoptosis triggered by bupivacaine and high glucose. In conclusion, our data indicated that local anesthetics may aggravate nerve toxicity in a high-glucose environment.

## 1. Introduction

Diabetic nerves are more susceptible to the toxicity of local anesthetics [1]. Clinical and experimental evidence has suggested that local anesthetics induced oxidative damage, which leads to neurotoxicity and apoptosis [2–4]. However, the mechanism of enhanced local anesthetic neurotoxicity under high-glucose conditions has not yet been fully understood. Diabetes can promote oxidative stress in some organs [5]. The reactive oxygen species (ROS) overexpression under hyperglycemia conditions has involved multiple pathways. For example, redox imbalances caused by upregulated aldose reductase activity, altered activity of protein kinase C, elevated advanced glycation end products, and prostanoid imbalances may lead to ROS overproduction under hyperglycemia conditions [6, 7]. ROS overproduction causes DNA degradation and induces neuronal apoptosis [8]. The accumulating studies have suggested that damaged nucleic acids were found in the certain tissues of diabetic rats [9, 10]. The current study aims to address whether ROS-mediated DNA damage aggrandized bupivacaine-induced neurotoxicity under high-glucose conditions.

DNA repair is critical for cell survival and normal cellular functions [11]. Chromosomal double-strand breaks jeopardize genome integrity as aberrant repair may cause genome rearrangements [12]. Nonhomologous end joining is predominantly responsible for double-strand break repair in mammals [13]. Ku70 plays a key role in the nonhomologous end-joining process. The Ku70 protein expresses ubiquitously in mammalian cells and localizes to both the cytosol and the nucleus [14]. It can bind to the DNA double-strand breaks, activate the DNA-activated protein kinase (DNA-PK), and then initiate the repair process [5]. Therefore, Ku70 is essential for nonhomologous double-strand break repair [5]. Intriguingly, chronic hyperglycemia suppresses the nuclear Ku70 expression in pancreatic acinar AR42J cells [5], but its impact on the Ku70 expression following nerve block anesthesia-induced DNA damage still remains unknown.

In the current study, we overexpressed Ku70 in SH-SY5Y cells to investigate whether the Ku70 expression inhibited by hyperglycemia suppressed the DNA damage repair and increased the damage induced by bupivacaine.

## 2. Materials and Methods

### 2.1. Cell Culture and Lentiviral Infection

SH-SY5Y and HEK293T cells from the Shanghai Institutes for Biological Sciences were maintained in Dulbecco's modified Eagle's medium (DMEM, Gibco, Carlsbad, CA, USA) containing 10% fetal bovine serum (Gibco), 100 IU/ml penicillin, and 100 mg/ml streptomycin (Gibco). The cells were grown in a humidified incubator (37°C; 5% CO_2_), with medium renewal every 2 days. The cells within 10 passages were used for experiments.

The full-length human Ku70 cDNA was inserted into the lentiviral vector LV5 (GenePharma Co., Ltd., Shanghai, China) and confirmed by sequencing. Lentiviral constructs of the LV5 empty vector or LV5-Ku70 were cotransfected with viral packaging plasmids (pGag/pol, pRev, and pVSV-G) into HEK293T cells by RNAi-mate (GenePharma) based on manufacturers' instructions. The viral supernatant was collected 72 h following transfection, which was filtered through a 0.45 *μ*m filter. Ku70-expressing lentivirus (Ku70+) or control lentivirus (LV5) was transfected into SH-SY5Y cells with 5 *μ*g/ml Polybrene. The Ku70 expression was confirmed by RT-PCR and western blotting.

### 2.2. MTT Assay

The MTT assay was conducted to evaluate the cell viability. The cells were grown in 96-well plates (1.0 × 10^4^/well). After a 24-hour starvation with the serum-free DMEM medium, the cells were subjected to bupivacaine hydrochloride treatment (0.5, 1.0, 1.5, 2.0, and 2.5 mM; Sigma Aldrich, St. Louis, MO, USA) for another 24 h, followed by the incubation with MTT (3-(4,5-dimethylthiazol-2-yl)-2,5-diphenyltetrazolium bromide; Beyotime, China) at 37°C for 4 hours. Upon MTT removal, 150 *μ*l DMSO was applied to each well, after which the resulting purple formazan was determined by the spectrophotometer (PowerWave™, Bio-Tek, Vermont, USA). All experimental groups were calculated as percentage of the control group with no treatments.

### 2.3. Quantitative RT-PCR (qRT-PCR)

Total RNA extraction was conducted using TRIzol (Invitrogen, Carlsbad, CA, USA) based on manufacturers' specifications. Using the M-MLV RT kit (Promega, Madison, WI, USA), 2 *μ*g RNA was reverse-transcribed into cDNA. The PCR reaction using resultant cDNA was conducted using the following primers: Ku70: 5′-GTGGTCACACACGAGCTTATT-3′ (sense) and 5′-CAAATGTCTGATGTTGGTGAACC-3′ (antisense) and *β*-actin: 5′-TGGATCAGCAAGCAGGAGTA-3′ (sense) and 5′-TCGGCCACATTGTGAACTTT-3′ (antisense). The thermal cycle for PCR was set as follows: 95°C for 2 min, 30 cycles of 94°C for 30 s, 56°C for 45 s, and 72°C for 45 s, with a final extension at 72°C for 7 min on a Lightcycler 480 system (Applied Biosystems, Foster City, CA, USA) using the SYBR Green Master Mixes (Takara, Japan). The Ku70 expression level was normalized to *β*-actin followed by the quantification through the 2^−ΔΔCt^ method [15].

### 2.4. Western Blotting

SH-SY5Y cells were lysed in the RIPA buffer as previously described [16]. After centrifugation (12,000*g*; 5 min), the supernatant was collected, mixed with the Laemmli buffer, and boiled for 10 min. Based on the protein concentration measured by the bicinchoninic acid (BCA) protein assay, 20 *μ*g protein was separated by sodium dodecyl sulfate-polyacrylamide gel electrophoresis and electrotransferred to polyvinylidene fluoride membranes. After blocking with 5% nonfat milk, primary antibodies, polyclonal anti-Ku70 (1 : 1,000; Novus Biologicals, Littleton, CO), anti-cleaved caspase-3 (1 : 1,000; Cell Signaling Technology, Danvers, MA, USA), and anti-actin antibody (1 :1,000; Cell Signaling Technology) were applied for overnight incubation at 4°C. After that, horseradish peroxidase-conjugated anti-rabbit immunoglobulin (1 : 1,000; Bioteke, Beijing, China) was applied to the membrane for a 1-hour incubation. The membrane was then developed using enhanced chemiluminescence (ECL; Takara Bio, Japan). The expression of the Ku70 protein or cleaved caspase-3 protein was quantified through measuring the band density using NIH Image J software (National Institutes of Health, Bethesda, MD, USA) and normalized to *β*-actin.

### 2.5. Experimental Grouping

Neuroblasts were divided into six groups which varied with or without receiving sequential treatments of 50 mM glucose (Sigma Aldrich) for 7 d (HG), infection with control lentivirus (LV5) or Ku70-expressing lentivirus (Ku70+), and then 1.0 mM bupivacaine for 24 h (Bup): Group a (control), serum-free DMEM medium; Group b (HG); Group c (Bup); Group d (HG + Bup); Group e (LV + HG + Bup); and Group f (LV-Ku70 + HG + Bup). An equal amount of the serum-free DMEM medium was added when there was no glucose or bupivacaine treatment.

### 2.6. ROS Measurement

The cells were stained with 20 *μ*M DCFH-DA (2′,7′-dichlorofluorescein diacetate; Molecular Probes, Eugene, OR, USA) at 37°C for 20 min. During this process, ROS oxidized DCFH-DA and produced highly fluorescent dichlorofluorescein (DCF). The fluorescence signals within the cells were read by an FAC Sort analyzer (Becton Dickinson, San Jose, CA, USA) and analyzed by Cell Quest software (Becton Dickinson).

### 2.7. Comet Assay

Single-cell gel electrophoresis (SCGE) was conducted to evaluate nucleic acid damages following treatment as previously described [17–19]. In brief, the cells were resuspended in 75 *μ*l of 0.5% low-melting agarose in PBS, immediately spread over agarose-coated slides, covered with a cover slip, and kept at 4°C for 10 min. The cover slip was removed after the solidification process. The cells were lysed by placing the slide into cold lysis solution (2.5 M NaCl, 100 mM Na_2_EDTA, 10 mM Tris, and 1% sodium sarcosinate, pH 10.0) with 1% Triton X-100 and 10% DMSO added before use. The lysis process was performed at least one hour at 4°C. Then, the slides were placed into the fresh electrophoresis buffer (1 mM Na_2_EDTA and 300 mM NaOH, pH 13.0) for 40 min for DNA unwinding and alkali-labile damage expression. The samples were subjected to electrophoresis (1.6 V cm^−1^; 300 mA; 20 min), followed by washings with the neutralizing buffer (0.4 M Tris, pH 7.5) three times. After that, the slides were stained with ethidium bromide (20 *μ*g/ml) for 10 min, and the comets were analyzed using inverted microscopy (Nikon Eclipse TE300, Japan) at 200x magnification. The CoolSNAP CCD camera (Photometrics, Tucson, AZ) was used to acquire images. Fifty cells per slide were scored in triplicate using CASP (Comet Assay Software Project) 6.0 software (University of Wroclaw, Poland). DNA damage is shown as the Olive tail moment given by

(1) $$\begin{array}{c} \text{Olive tail moment}=\left(\text{optical density barycenter in the tail}-\text{optical density barycenter in the head}\right)\times \text{percent tail DNA}. \end{array}$$

### 2.8. Cell Apoptosis Analysis

The cells were plated in 24-well plates (5 × 10^5^ cells/well; 500 *μ*l) and stimulated as described. After rinsing with PBS, the cells were collected and resuspended in the binding buffer, with annexin V-FITC (1 : 100; KeyGEN, Nanjing, China) and propidium iodide (PI; 1 : 100; KeyGEN) applied for staining. Following 10 min incubation, the cells were examined via flow cytometry, with early apoptotic cells being annexin V-FITC-positive and PI-negative.

### 2.9. TUNEL Assay

Following the fixation by 4% formaldehyde at 4°C for 25 min, the cells were rinsed with PBS and then incubated with 0.2% Triton X-100 at room temperature for 5 min. After equilibration, the TdT labeling reaction mixture was applied to the section for a 60 min incubation at 37°C. The apoptotic ratio was calculated by counting 100 cells in 3 randomly selected fields via fluorescent microscopy (AX-80, Olympus, Tokyo, Japan) by two independent researchers.

### 2.10. Statistical Analysis

Data were shown as mean ± standard deviation (SD). IBM SPSS Statistics Software Version 20 (IBM, San Francisco, CA, USA) was used to obtain statistics. Two-tailed, unpaired Student's *t*-test was conducted for comparisons between two groups, while one-way analysis of variance (ANOVA) followed by an LSD post hoc test was employed for comparisons among three or more groups. A *P* value less than 0.05 was set as statistically significant.

## 3. Results

### 3.1. Bupivacaine-Induced Cytotoxicity

**Figure 1:**
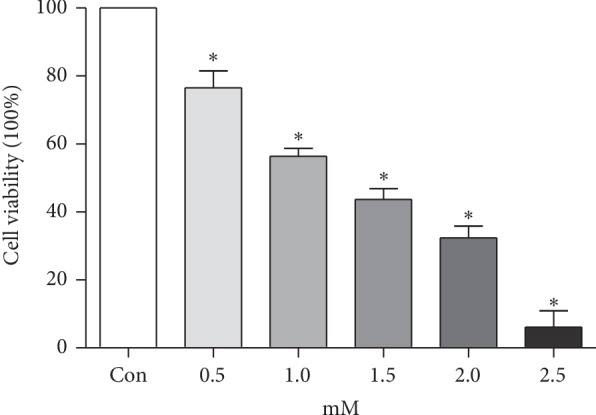
Cell viability declined in a bupivacaine concentration-dependent manner. After serum starvation in the DMEM/F12 medium for 24 h, the cells were exposed to 0.5, 1.0, 1.5, 2.0, and 2.5 mM bupivacaine for 24 h. Values are the mean ± SEM of *n* = 3. ^*∗*^*P* < 0.05 compared with the untreated control.

To evaluate the bupivacaine-induced cytotoxicity in SH-SY5Y cells, the MTT assay was conducted after a 24 h exposure to various doses of bupivacaine (0.5–2.5 mM) according to previous references [3, 20–22]. We found that cell viability was dramatically decreased in a dose-dependent fashion (Figure 1). More than 60% reduction was observed with 2.0 and 2.5 mM bupivacaine. To better understand the mechanism underlying bupivacaine-induced neurotoxicity and identify the key contributors to this process, the concentrations of 0.5, 1.0, and 1.5 mM were used in the following experiments.

### 3.2. Expression of Ku70 and Cleaved Capsase-3 upon Bupivacaine Treatment

**Figure 2:**
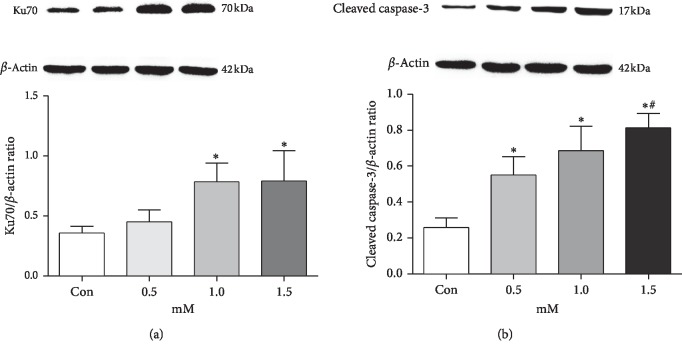
Repair protein Ku70 expression was elevated by bupivacaine. (a) Western blotting bands and data of the repair protein Ku70 expression. (b) Western blotting bands and data of the cleaved caspase-3 expression. SH-SY5Y cells' serum starved for 24 h followed by incubation with 0.5, 1.0, or 1.5 mM bupivacaine for 24 h. Values are the mean ± SEM of *n* = 3. ^*∗*^*P* < 0.05 compared with the untreated control ^*#*^*P* < 0.05 compared with the group incubated with 1.0 mM bupivacaine.

To determine the effect of bupivacaine on the expression of Ku70 and cleaved caspase-3, their protein levels were detected in SH-SY5Y cells after a 24-hour stimulation by increasing doses of bupivacaine (0.5, 1.0, and 1.5 mM) by western blotting. The elevation in their expression was also dose-dependent, which paralleled the bupivacaine-induced cell damage result. These results indicated that the Ku70 expression and cell apoptosis were enhanced in response to bupivacaine exposure (Figure 2). The concentration of 1.0 mM had a similar effect on Ku70 protein levels as that of 1.5 mM. Thus, we chose a level of 1.0 mM for the following studies.

### 3.3. Expression of Ku70 Is Inhibited by Long-Term High-Glucose Treatment

**Figure 3:**
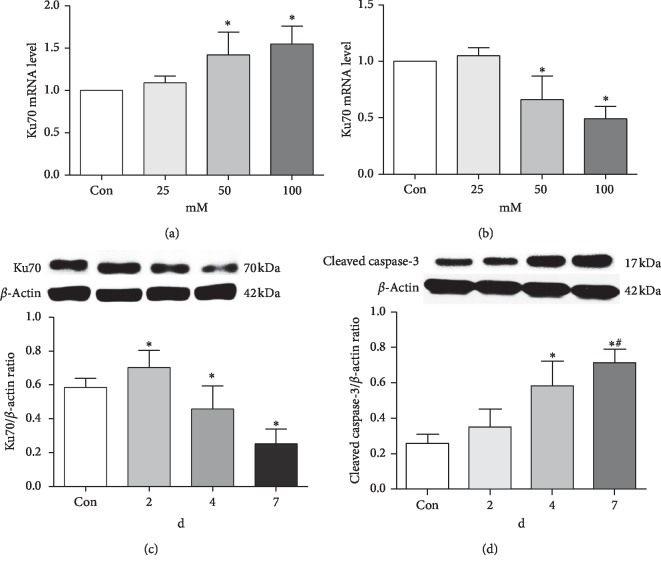
Long-term high-glucose conditions inhibited the Ku70 expression and induced more severe apoptosis. (a) Ku70 mRNA levels in SH-SY5Y cells' serum starved for 24 h followed by incubation with increasing glucose concentrations (25, 50, or 100 mM) for 2 days. (b) Ku70 mRNA levels in SH-SY5Y cells' serum starved for 24 h followed by incubation with increasing glucose concentrations (25, 50, or 100 mM) for 7 days. (c) Western blotting bands and data of the Ku70 expression in SH-SY5Y cells' serum starved for 24 h and were then incubated with 50 mM glucose for 2, 4, or 7 days. (d) Western blotting bands and data of the cleaved caspase-3 expression in SH-SY5Y cells' serum starved for 24 h and were then incubated with 50 mM glucose for 2, 4, or 7 days. Values are the mean ± SEM of *n* = 3. ^*∗*^*P* < 0.05 compared with the untreated control. ^#^*P* < 0.05 compared with the group incubated with 50 mM glucose for 4 days.

To determine the effect of high glucose on the Ku70 expression, Ku70 mRNA levels were detected in SH-SY5Y cells stimulated by various concentrations of glucose for 2 or 7 days. As previously described, 25 mM of glucose represents normal plasma glucose [7, 23]. Here, 25, 50, and 100 mM of glucose were used. Our results have shown a significant elevation in the Ku70 mRNA expression after 2 d of high-glucose exposure (50 and 100 mM) (Figure 3(a)). Nevertheless, the cells exhibited lower Ku70 mRNA expression after the treatment with higher concentrations of glucose (50 or 100 mM) for 7 d (Figure 3(b)). There was no significant difference between the cells exposed to 50 and 100 mM of glucose. Thus, 50 mM was chosen for the following experiments. We also investigated the protein levels of Ku70 in the cells stimulated with 50 mM glucose for 2, 4, or 7 days and found that the Ku70 level was elevated after 2 days but decreased after 4 and 7 days (Figure 3(c)). The cleaved caspase-3 levels were increased by 50 mM glucose in a time-dependent fashion (Figure 3(d)). In conclusion, high glucose (50 mM) suppressed the Ku70 expression, which may cause insufficient repair of DNA damage and ultimately cell apoptosis.

### 3.4. Protein Levels of Ku70 and Cleaved Capsase-3 in response to Bupivacaine Exposure under a High-Glucose Environment

**Figure 4:**
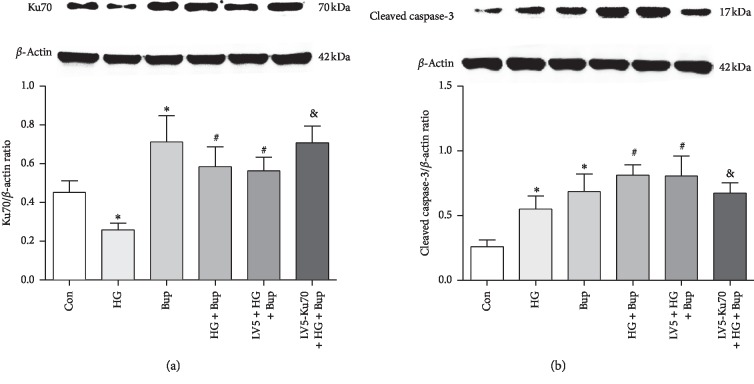
High glucose inhibited the Ku70 expression and enhanced bupivacaine-induced apoptosis. (a) Western blotting bands and data of the Ku70 expression in SH-SY5Y cells. (b) Western blotting bands and data of the cleaved caspase-3 expression in SH-SY5Y cells. Con: untreated SH-SY5Y cells; HG: SH-SY5Y cells exposed to 50 mM glucose for 7 days; Bup: SH-SY5Y cells treated with 1.0 mM bupivacaine for 24 h; HG + Bup: SH-SY5Y cells incubated with 50 mM glucose for 7 days before treatment with 1.0 mM bupivacaine for 24 h; LV5 + HG + Bup: SH-SY5Y cells transfected vector (LV5) incubated with 50 mM glucose for 7 days before treatment with 1.0 mM bupivacaine for 24 h; LV5-Ku70 + HG + Bup: SH-SY5Y cells transfected overexpression lentivirus (LV5-Ku70) incubated with 50 mM glucose for 7 days before treatment with 1.0 mM bupivacaine for 24 h. Values are the mean ± SEM of *n* = 3. ^*∗*^*P* < 0.05 compared with Con. ^#^*P* < 0.05 compared with HG and Bup. ^&^*P* < 0.05 compared with HG + Bup and LV5 + HG + Bup.

We then tried to determine the effects of bupivacaine-combined high-glucose treatment on the protein levels of Ku70 and cleaved caspase-3. Our results have shown that Ku70 protein levels were significantly elevated by bupivacaine treatment in SH-SY5Y cells, which were remarkably decreased by pretreatment with high glucose for 7 days (Figure 4(a)). Moreover, infection of the Ku70-expressing virus significantly increased the Ku70 expression compared to control virus infection under normal conditions (Figures [Supplementary-material supplementary-material-1] and [Supplementary-material supplementary-material-1]) or high-glucose conditions following bupivacaine exposure (Figure 4(b)). The cleaved caspase-3 expression was elevated in the cells pretreated with high glucose for 7 days and then exposed to bupivacaine for 24 h in comparison with those exposed to bupivacaine only. Infection of the Ku70-expressing virus significantly suppressed the levels of cleaved caspase-3 both under normal conditions ([Supplementary-material supplementary-material-1]) and in high-glucose- and bupivacaine-treated cells (Figure 4(b)).

### 3.5. High Glucose Enhances the Effects of Bupivacaine on ROS Production

**Figure 5:**
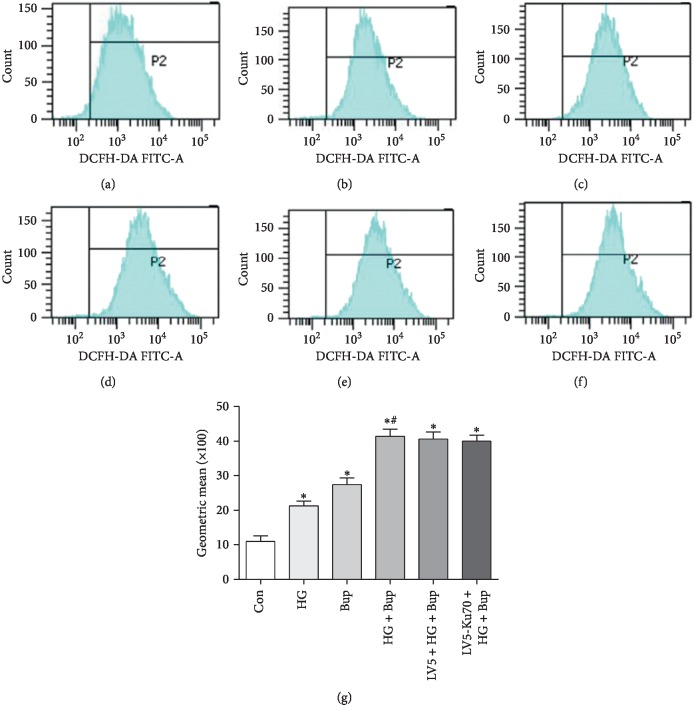
High glucose enhanced bupivacaine-induced ROS production. The ROS production as detected by redox-sensitive fluorescent dye DCFH-DA. Summarized data show the intracellular ROS content, expressed as the ratio of mean fluorescence intensity of treated groups to that of untreated controls. (a) Con: untreated SH-SY5Y cells. (b) HG: SH-SY5Y cells exposed to 50 mM glucose for 7 days. (c) Bup: SH-SY5Y cells treated with 1.0 mM bupivacaine for 24 h. (d) HG + Bup: SH-SY5Y cells incubated with 50 mM glucose for 7 days before treatment with 1.0 mM bupivacaine for 24 h. (e) LV5 + HG + Bup: SH-SY5Y cells transfected vector (LV5) incubated with 50 mM glucose for 7 days before treatment with 1.0 mM bupivacaine for 24 h. (f) LV5-Ku70 + HG + Bup: SH-SY5Y cells transfected overexpression lentivirus (LV5-Ku70) incubated with 50 mM glucose for 7 days before treatment with 1.0 mM bupivacaine for 24 h. (g) Values are the mean ± SEM of *n* = 6. ^*∗*^*P* < 0.05 compared with Con. ^#^*P* < 0.05 compared with HG and Bup.

The cells exhibited elevated intracellular ROS with 50 mM glucose or 1 mM bupivacaine stimulation (Figure 5). Pretreatment with glucose induced even significantly higher ROS levels, implying that high glucose enhanced the induction effects of bupivacaine on intracellular ROS production. No significant difference was found in ROS production between the Ku70 virus infection group and the control virus infection group.

### 3.6. Synergistic Effect of High Glucose and Bupivacaine on DNA Damage and Cell Apoptosis

**Figure 6:**
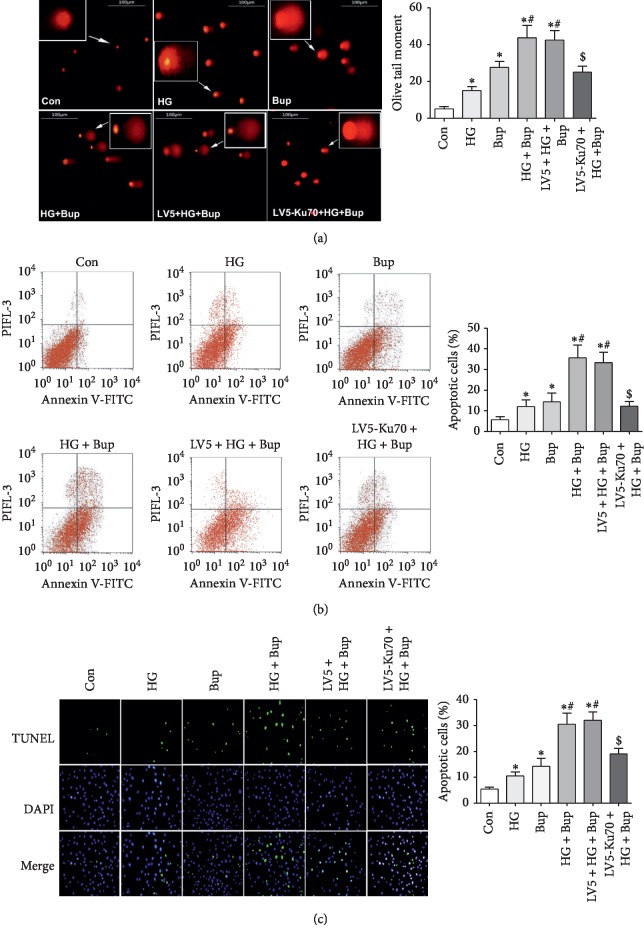
High glucose enhanced bupivacaine-induced DNA damage and cell apoptosis, which was attenuated by the Ku70 overexpression. (a) DNA damage as detected by the cosmet assay and the Olive tail moment. (b) Cells in early apoptosis are annexin V-FITC-positive (right upper quadrant) and PI-negative (right lower quadrant). Summarized data show the early apoptotic rates as detected by flow cytometry. (c) Cell apoptosis as detected by the TUNEL assay. Con: untreated SH-SY5Y cells; HG: SH-SY5Y cells exposed to 50 mM glucose for 7 days; Bup: SH-SY5Y cells treated with 1.0 mM bupivacaine for 24 h; HG + Bup: SH-SY5Y cells incubated with 50 mM glucose for 7 days before treatment with 1.0 mM bupivacaine for 24 h; LV5 + HG + Bup: SH-SY5Y cells transfected vector (LV5) incubated with 50 mM glucose for 7 days before treatment with 1.0 mM bupivacaine for 24 h; LV5-Ku70 + HG + Bup: SH-SY5Y cells transfected overexpression lentivirus (LV5-Ku70) incubated with 50 mM glucose for 7 days before treatment with 1.0 mM bupivacaine for 24 h. Values are the mean ± SEM of *n* = 3. ^*∗*^*P* < 0.05 compared with Con. ^#^*P* < 0.05 compared with HG and Bup. ^&^*P* < 0.05 compared with HG + Bup and LV5 + HG + Bup.

We further quantified DNA damage by employing the comet assay as previously described [18]. Olive tail moment values were increased by bupivacaine, which was synergistic with the high-glucose condition. The ectopic expression of Ku70 significantly reduced Olive tail moment values in the cells stimulated by high glucose and bupivacaine (Figure 6(a)).

We then evaluated cell apoptosis by annexin V/PI staining and TUNEL assay. There was an increase in annexin V-FITC-positive and PI-negative cells upon 50 mM glucose treatment, indicating 50 mM glucose promoted bupivacaine-induced apoptosis (Figure 6(b)). The overexpression of Ku70 reduced bupivacaine-induced apoptosis in a high-glucose environment. This was further supported by the results of TUNEL staining (Figure 6(c)).

## 4. Discussion

Neuroblastoma SH-SY5Y cell line, as the only available cell line with the features of healthy spinal dorsal root ganglion cells [24], has been commonly used to establish *in vitro* models for studying cellular functions of neuronal cells [21], including our study here.

Regional anesthesia may increase the risk of neurological injury in diabetic neuropathy patients [25, 26]. Diabetes mellitus is characterized by hyperglycemia, resulting in excess mitochondrial ROS generation [27]. Dosing of lidocaine in diabetic animals induced ROS generation, which was suggested to trigger oxidative stress and apoptosis [1]. Understanding the underlying mechanisms of neurotoxicity will shed light on the clinical nerve blockade in patients with hyperglycemia and may offer potential pharmacological interventions. This study proved a high degree of ROS generation in SH-SY5Y cells stimulated by high glucose and bupivacaine.

Oxidative stress is characterized by overwhelming ROS-induced DNA damage [28]. Double-strand breaks as the most serious DNA damage are identified and fixed by conserved repair pathways to preserve genomic integrity [29]. Improper DNA repair may cause damage accumulation, eventually leading to cell apoptosis or diseases. After the failure of repair, ROS production triggered neuronal cell apoptosis via releasing cytochrome c from mitochondria and cleaved caspase-3 [30].

Despite the importance of homologous recombination, nonhomologous end joining is predominantly responsible for repairing double-strand breaks in mammals [31]. The main proteins involved in the classic nonhomologous end joining include Ku70/Ku80 heterodimer, DNA-activated protein kinase catalytic subunit (DNA-PKcs), Artemis, and XRCC4 [32]. The nonhomologous end-joining process usually takes three steps: recognition of the DNA breaks, removal of the broken ends, and ligation of the DNA terminals. Ku70 has been reported to play a key role during nonhomologous end joining as a 5′-deoxyribose-5-phosphate/apurinic site lyase (5′-dRP/AP lyase). Specifically, DNA repair starts from the premise that the abasic and apurinic sites are excised by Ku70. It not only prevents the activation of endonucleases but also protects the broken DNA ends [33].

Ku70 binds to the broken DNA ends and initiates the nonhomologous end-joining process through a two-step mechanism, where Ku70 identifies DNA ends, translocates to internal positions, recruits DNA-PKcs, and forms DNA-PK holoenzyme [34, 35]. Ku70 regulates the DNA-PK activity either by recruiting DNA-PKcs to DNA at low DNA end concentration or by direct protein-protein interactions [36]. Ku70 may involve in the oxidative stress-induced cell death. Moreover, Ku70 reduction has been shown to contribute to cell apoptosis. Specifically, the blockage of the Ku70 expression induces the apoptosis of human promyelocytic leukemia HL-60 cells and activates human peripheral blood lymphocytes [37]. Here, bupivacaine caused double-strand breaks in SH-SY5Y cells, which induces the expression of Ku70, an efficient DNA repair machinery to cope with DNA damages.

High glucose may downregulate the synthesis of DNA repair protein Ku70 through inhibition of its translation and/or posttranslational protein modification [38]. Here, the high-glucose environment aggravated the effects of bupivacaine on DNA damage and cell apoptosis, which was mitigated by the overexpression of Ku70. These data suggested that Ku70 was a key regulator in bupivacaine-induced double-strand breaks and cell apoptosis in a high-glucose environment. In this study, a high level of ROS was detected in SH-SY5Y cells induced by bupivacaine in a high-glucose environment, which was not affected by the Ku70 overexpression, indicating Ku70 may not involve in the high-glucose- and bupivacaine-induced ROS generation.

In the current study, most experiments were performed with 0.5, 1.0, and 1.5 mM bupivacaine. The concentration was chosen according to previous studies [4–7] and MTT assays (Figure 1). After epidural or spinal administration with 0.5% bupivacaine, the serum concentration of bupivacaine in patients [39, 40] and experimental animals [41] was about 1.25∼1.6 *μ*g/ml, which equaled 3.65∼4.67 × 10^−3^ mM and was much lower than that in in vitro experiments. Therefore, to verify the function of Ku70 in vivo, experiments using animal models treated with clinical concentrations of bupivacaine will be performed in the future.

In summary, our study with SH-SY5Y cells indicated the synergistic effect of high glucose and bupivacaine on intracellular ROS and the aggravated effect on apoptosis, which proceeded via Ku70 suppression and caspase-3 activation. Thus, the present data provide a possible mechanistic basis for aggravated effects of local anesthetics on high-glucose-induced nerve toxicity.

## Data Availability

The data used to support the findings of this study are available from the corresponding author upon request.

## Supplementary Materials

Supplementary Materials Figure S1: Ku70 overexpression lentivirus increased the expression of Ku70 and inhibited the expression of cleaved caspase-3 in SH-SY5Y cells.

## References

[1] Lirk P., Flatz M., Haller I. (2012). In zucker diabetic fatty rats, subclinical diabetic neuropathy increases in vivo lidocaine block duration but not in vitro neurotoxicity. *Regional Anesthesia and Pain Medicine*.

[2] Perez-Castro R., Patel S., Garavito-Aguilar Z. V. (2009). Cytotoxicity of local anesthetics in human neuronal cells. *Anesthesia & Analgesia*.

[3] Lu J., Xu S. Y., Zhang Q. G., Lei H. Y. (2011). Bupivacaine induces reactive oxygen species production via activation of the AMP-activated protein kinase-dependent pathway. *Pharmacology*.

[4] Park C. J., Park S. A., Yoon T. G., Lee S. J., Yum K. W., Kim H. J. (2005). Bupivacaine induces apoptosis via ROS in the schwann cell line. *Journal of Dental Research*.

[5] Song J. Y., Lim J. W., Kim H., Morio T., Kim K. H. (2003). Oxidative stress induces nuclear loss of DNA repair proteins Ku70 and Ku80 and apoptosis in pancreatic acinar AR42J cells. *Journal of Biological Chemistry*.

[6] Baynes J. W., Thorpe S. R. (1999). Role of oxidative stress in diabetic complications: a new perspective on an old paradigm. *Diabetes*.

[7] Russell J. W., Sullivan K. A., Windebank A. J., Herrmann D. N., Feldman E. L. (1999). Neurons undergo apoptosis in animal and cell culture models of diabetes. *Neurobiology of Disease*.

[8] Sullivan K. A., Hayes J. M., Wiggin T. D. (2007). Mouse models of diabetic neuropathy. *Neurobiology of Disease*.

[9] Puthanveetil P., Zhang D., Wang Y. (2012). Diabetes triggers a PARP1 mediated death pathway in the heart through participation of FoxO1. *Journal of Molecular and Cellular Cardiology*.

[10] Kilarkaje N., Al-Bader M. M. (2015). Diabetes-induced oxidative DNA damage alters p53-p21CIP1/Waf1 signaling in the rat testis. *Reproductive Sciences*.

[11] Wang G., Vasquez K. M. (2014). Impact of alternative DNA structures on DNA damage, DNA repair, and genetic instability. *DNA Repair*.

[12] So E. Y., Ouchi T. (2014). Decreased DNA repair activity in bone marrow due to low expression of DNA damage repair proteins. *Cancer Biology & Therapy*.

[13] Sung P. A., Libura J., Richardson C. (2006). Etoposide and illegitimate DNA double-strand break repair in the generation of MLL translocations: new insights and new questions. *DNA Repair*.

[14] Sawada M., Sun W., Hayes P., Leskov K., Boothman D. A., Matsuyama S. (2003). Ku70 suppresses the apoptotic translocation of Bax to mitochondria. *Nature Cell Biology*.

[15] Inturi S., Tewari-Singh N., Agarwal C., White C. W., Agarwal R. (2014). Activation of DNA damage repair pathways in response to nitrogen mustard-induced DNA damage and toxicity in skin keratinocytes. *Mutation Research/Fundamental and Molecular Mechanisms of Mutagenesis*.

[16] Ayene I. S., Ford L. P., Koch C. J. (2005). Ku protein targeting by Ku70 small interfering RNA enhances human cancer cell response to topoisomerase II inhibitor and *γ* radiation. *Molecular Cancer Therapeutics*.

[17] Singh N. P., McCoy M. T., Tice R. R., Schneider E. L. (1988). A simple technique for quantitation of low levels of DNA damage in individual cells. *Experimental Cell Research*.

[18] Inturi S., Tewari-Singh N., Gu M. (2011). Mechanisms of sulfur mustard analog 2-chloroethyl ethyl sulfide-induced DNA damage in skin epidermal cells and fibroblasts. *Free Radical Biology and Medicine*.

[19] Tice R. R., Agurell E., Anderson D. (2000). Single cell gel/comet assay: guidelines for in vitro and in vivo genetic toxicology testing. *Environmental and Molecular Mutagenesis*.

[20] Li Y.-J., Zhao W., Yu X.-J. (2017). Activation of p47phox as a mechanism of bupivacaine-induced burst production of reactive oxygen species and neural toxicity. *Oxidative Medicine and Cellular Longevity*.

[21] Li L., Ye X.-P., Lu A.-Z. (2013). Hyperglycemia magnifies bupivacaine-induced cell apoptosis triggered by mitochondria dysfunction and endoplasmic reticulum stress. *Journal of Neuroscience Research*.

[22] Wen X., Xu S., Liu H. (2013). Neurotoxicity induced by bupivacaine via T-type calcium channels in SH-SY5Y cells. *PLoS One*.

[23] Russell J. W., Golovoy D., Vincent A. M. (2002). High glucose-induced oxidative stress and mitochondrial dysfunction in neurons. *The FASEB Journal*.

[24] Shang Y., Guo X. X., Li W. W. (2014). Cucurbitacin-B inhibits neuroblastoma cell proliferation through up-regulation of PTEN. *European Review for Medical and Pharmacological Sciences*.

[25] Lirk P., Verhamme C., Boeckh R. (2014). Effects of early and late diabetic neuropathy on sciatic nerve block duration and neurotoxicity in Zucker diabetic fatty rats. *British Journal of Anaesthesia*.

[26] Lirk P., Birmingham B., Hogan Q. (2011). Regional anesthesia in patients with preexisting neuropathy. *International Anesthesiology Clinics*.

[27] West I. C. (2000). Radicals and oxidative stress in diabetes. *Diabetic Medicine*.

[28] Beckman K. B., Ames B. N. (1998). The free radical theory of aging matures. *Physiological Reviews*.

[29] Stevens M. J., Obrosova I., Cao X., Van Huysen C., Greene D. A. (2000). Effects of DL-alpha-lipoic acid on peripheral nerve conduction, blood flow, energy metabolism, and oxidative stress in experimental diabetic neuropathy. *Diabetes*.

[30] Annunziato L., Amoroso S., Pannaccione A. (2003). Apoptosis induced in neuronal cells by oxidative stress: role played by caspases and intracellular calcium ions. *Toxicology Letters*.

[31] Lieber M. R., Ma Y., Pannicke U., Schwarz K. (2003). Mechanism and regulation of human non-homologous DNA end-joining. *Nature Reviews Molecular Cell Biology*.

[32] Dobbs T. A., Tainer J. A., Lees-Miller S. P. (2010). A structural model for regulation of NHEJ by DNA-PKcs autophosphorylation. *DNA Repair*.

[33] Roberts S. A., Strande N., Burkhalter M. D. (2010). Ku is a 5′-dRP/AP lyase that excises nucleotide damage near broken ends. *Nature*.

[34] Tuteja R., Tuteja N. (2000). Ku autoantigen: a multifunctional DNA-binding protein. *Critical Reviews in Biochemistry and Molecular Biology*.

[35] Ramsden D. A., Gellert M. (1995). Formation and resolution of double-strand break intermediates in V(D)J rearrangement. *Genes & Development*.

[36] Sawada M., Hayes P., Matsuyama S. (2003). Cytoprotective membrane-permeable peptides designed from the Bax-binding domain of Ku70. *Nature Cell Biology*.

[37] Ajmani A. K., Satoh M., Reap E., Cohen P. L., Reeves W. H. (1995). Absence of autoantigen Ku in mature human neutrophils and human promyelocytic leukemia line (HL-60) cells and lymphocytes undergoing apoptosis. *The Journal of Experimental Medicine*.

[38] Li J., Ayene R., Ward K. M., Dayanandam E., Ayene I. S. (2009). Glucose deprivation increases nuclear DNA repair protein Ku and resistance to radiation induced oxidative stress in human cancer cells. *Cell Biochemistry and Function*.

[39] Kaukinen L., Kaukinen S., Karkkainen S. (1986). Epidural anesthesia with carticaine in cesarean section: a comparison with bupivacaine. *Regional-Anaesthesie*.

[40] Cheung S. L., Booker P. D., Franks R., Pozzi M. (1997). Serum concentrations of bupivacaine during prolonged continuous paravertebral infusion in young infants. *British Journal of Anaesthesia*.

[41] Shilo-Benjamini Y., Pypendop B. H., Newbold G., Pascoe P. J. (2017). Plasma bupivacaine concentrations following orbital injections in cats. *Veterinary Anaesthesia and Analgesia*.

