# Supplementary material for: Inhibition of DNA Repair Protein Ku70 in High-Glucose Environment Aggravates the Neurotoxicity Induced by Bupivacaine in SH-SY5Y Cells
Source: Biomed Res Int. 2020 Jan 31;2020:1283214. doi: 10.1155/2020/1283214 (PMC7013357; doi:10.1155/2020/1283214)
Supplement: Supplementary Materials — Figure S1: Ku70 overexpression lentivirus increased the expression of Ku70 and inhibited the expression of cleaved caspase-3 in SH-SY5Y cells. [file 1283214.f1.docx]

**
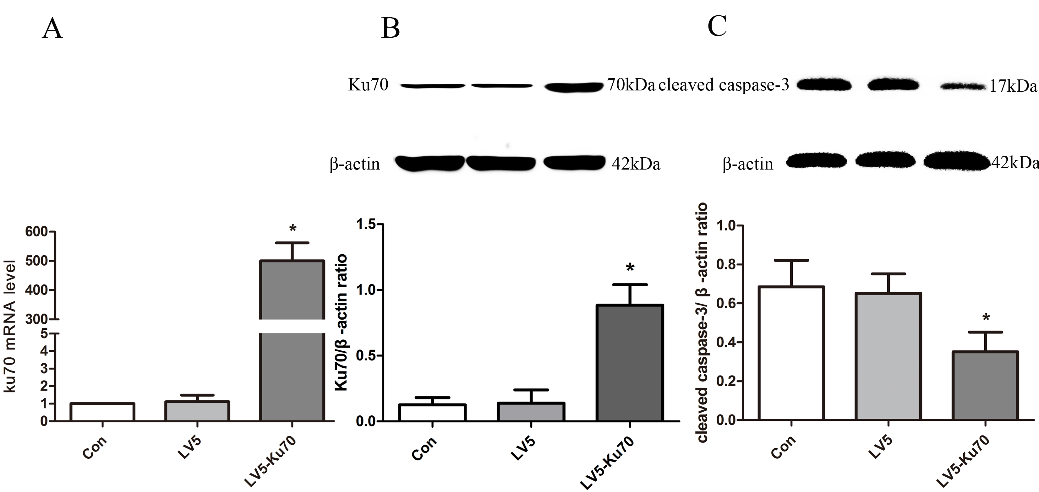
**

**Figure S1** Ku70 overexpession lentivirus increased the expression of Ku70 and inhibited the expression of cleaved caspase-3 in SH-SY5Y cells. (A) Ku70 mRNA levels in SH-SY5Y cells transfected vector (LV5) and overexpression lentivirus (LV5-Ku70). (B) The western blotting bands and data of Ku70 expression in SH-SY5Y cells transfected vector (LV5) and overexpression lentivirus (LV5-Ku70). (C) The western blotting bands and data of cleaved caspase-3 expression in SH-SY5Y cells transfected vector (LV5) and overexpression lentivirus (LV5-Ku70). Values are the mean ± SEM of n = 3, **P* < 0.05 compared with the untreated control.
